# Supplementary material for: Trehalose Production Using Three Extracellular Enzymes Produced via One-Step Fermentation of an Engineered Bacillus subtilis Strain
Source: Bioengineering (Basel). 2023 Aug 18;10(8):977. doi: 10.3390/bioengineering10080977 (PMC10451709; doi:10.3390/bioengineering10080977)
Supplement: Supplementary file 1 [file bioengineering-10-00977-s001.zip › bioengineering-2527908-supplementary.pdf]

Trehalose Production Using Three Extracellular Enzymes Produced via One-Step Fermentation of an Engineered *Bacillus subtilis* Strain

Xi Sun [1,†], Jun Yang [1,†], Xiaoping Fu [2,3,†], Xingya Zhao [4,5], Jie Zhen [4,5], Hui Song [2,3,4,5], Jianyong Xu [2,4,5], Hongchen Zheng [2,3,4,5,*] and Wenqin Bai [2,3,*]

[1] College of Biological Engineering, Tianjin Agricultural University, Tianjin 300384, China; sunxi@tjau.edu.cn (X.S.); yangjun@tib.cas.cn (J.Y.)
[2] National Center of Technology Innovation for Synthetic Biology, Tianjin Institute of Industrial Biotechnology, Chinese Academy of Sciences, Tianjin 300308, China; fu_xp@tib.cas.cn (X.F.); song_h@tib.cas.cn (H.S.); xu_jy@tib.cas.cn (J.X.)
[3] Key Laboratory of Engineering Biology for Low-Carbon Manufacturing, Tianjin Institute of Industrial Biotechnology, Chinese Academy of Sciences, Tianjin 300308, China
[4] Industrial Enzymes National Engineering Research Center, Tianjin Institute of Industrial Biotechnology, Chinese Academy of Sciences, Tianjin 300308, China; zhao_xy@tib.cas.cn (X.Z.); zhen_j@tib.cas.cn (J.Z.)
[5] Tianjin Key Laboratory for Industrial Biological Systems and Bioprocessing Engineering, Tianjin Institute of Industrial Biotechnology, Chinese Academy of Sciences, Tianjin 300308, China
* Correspondence: zheng_hc@tib.cas.cn (H.Z.); baiwq@tib.cas.cn (W.B.); Tel.: +86-022-84861933 (H.Z.)
† These authors contributed equally to this work.

Table S1. Strains and plasmids used in this study.

Strains and plasmids	description	source
**Strains**		
E. coli BL21(DE3) TrxB	F^- omp^T *hsdS*$_B$ *(r*$_B^-$*m*$_B^-$*) gal dcm trxB*15::kan (DE3)	Our lab.
B. subtilis SCK6	Erm^R, *his, nprR2, nprE18, ΔaprA3, ΔeglS102, ΔbglT/bglSRV, lacA::PxylA-comK*	Our lab.
E. coli DH5*α*	F$^-$, φ80, *lac*Z ΔM15, Δ (*lac*ZYA-argF) U169 *end*A1, *rec*A1, *hsd*R17(r$_k^-$, m$_k^+$) *sup*E44, λ$^-$, thi^{-1}, *gyr*A96, *rel*A1, *pho*A	Our lab.
**Plasmids**		
pMA05	Amp^r (*E. coli*), Kan^r (*B. subtilis*), P$_{hapII}$, *B. subtilis -E. coli shuttle plasmid*	Our lab.
pMC68	Amp^r (*E. coli*), Kan^r (*B. subtilis*), P$_{hapII}$, CBM68 structural domain, *B. subtilis -E. coli shuttle plasmid*	Our lab.
pHT43	Amp^r *(E. coli),* Chl^r *(B. subtilis), lacO, B. subtilis - E. coli shuttle plasmid*	Our lab.
pET32a	Amp^r, T7*lac* promoter, Trx•Tag™ (N), His•Tag (I,C) and S•Tag (I)	Our lab.
pET32a-ARS	pET32a containing the MTSase gene from *Arthrobacter ramosus* S34	In this work

pET32a-SAS	pET32a containing the MTSase gene from *Sulfolobus acidocaldarius* ATCC33909	In this work
pET32a-SSS	pET32a containing the MTSase gene from *Sulfolobus solfataricus* KM1	In this work
pET32a-ARH	pET32a containing the MTHase gene from *Arthrobacter ramosus* S34	In this work
pET32a-SAH	pET32a containing the MTHase gene from *Sulfolobus acidocaldarius* ATCC33909	In this work
pET32a-SSH	pET32a containing the MTHase gene from *Sulfolobus solfataricus* KM1	In this work
pMC68-ARS	pMC68 containing the MTSase gene from *Arthrobacter ramosus* S34	In this work
pMC68-ARH	pMC68 containing the MTHase gene from *Arthrobacter ramosus* S34	In this work
pHT43-C68-ARS	pMC68 containing CBM68 structural domain and the MTSase gene from *Arthrobacter ramosus* S34	In this work
pHT43-C68-ARH	pMC68 containing CBM68 structural domain and the MTHase gene from *Arthrobacter ramosus* S34	In this work
pDG1730-*amyE*-ARS	Bacillus subtilis integrative plasmid, Amp^r (*E. coli*), Spc^r (*B. subtilis*), upstream and downstream homologous genes containing *amyE* sites, the MTSase gene from *Arthrobacter ramosus* S34	In this work
pDG1730-*amyE*-ARH	Bacillus subtilis integrative plasmid, Amp^r (*E.coli*), Spc^r (*B. subtilis*), upstream and downstream homologous genes containing *amyE* sites, the MTHase gene from *Arthrobacter ramosus* S34	In this work
pDG1730-*nprB*-ARS	Bacillus subtilis integrative plasmid, Amp^r (*E. coli*), Spc^r (*B. subtilis*), upstream and downstream homologous genes containing *nprB* sites, the MTSase gene from *Arthrobacter ramosus* S34	In this work
pDG1730-*nprB*-ARH	Bacillus subtilis integrative plasmid, Amp^r (*E. coli*), Spc^r (*B. subtilis*), upstream and downstream homologous genes containing *nprB* sites, the MTHase gene from *Arthrobacter ramosus* S34	In this work
pDG1730-*ytxE*-ARS	Bacillus subtilis integrative plasmid, Amp^r (*E. coli*), Spc^r (*B. subtilis*), upstream and downstream homologous genes containing *ytxE* sites, the MTSase gene from *Arthrobacter ramosus* S34	In this work
pDG1730-*ytxE*-ARH	Bacillus subtilis integrative plasmid, Amp^r (*E. coli*), Spc^r (*B. subtilis*), upstream and downstream homologous genes containing *ytxE* sites, the MTHase gene from *Arthrobacter ramosus* S34	In this work

Table S2. Primers used in this study.

Primer name	Sequence (5'-3')
32a-F	GATCCGGCTGCTAACAAAGC
32a-R	CATGGCCTTGTCGTCGTC
32a-ARS-F	TACCGACGACGACGACAAGGCCATGGTTCCGGCAAGCACATATA GAC
32a-ARS-R	TTCGGGCTTTGTTAGCAGCCGGATCTTAGTGATGATGATGATGATG TGTTTCGAC
32a-SAS-F	TACCGACGACGACGACAAGGCCATGGTTATTAGCGCAACATATCG CCT
32a-SAS-R	TTCGGGCTTTGTTAGCAGCCGGATCTCAATGGTGGTGGTGGTGGT GCTCG
32a-SSS-F	TACCGACGACGACGACAAGGCCATGATGATCATTGGCACATATCG CC
32a-SSS-R	TTCGGGCTTTGTTAGCAGCCGGATCTTAGTGATGGTGGTGATGAT GTTCG
32a-ARH-F	TACCGACGACGACGACAAGGCCATGATGAATCGCAGATTTCCAG TTTGG
32a-ARH-R	TTCGGGCTTTGTTAGCAGCCGGATCTTAGTGATGGTGATGATGAT GTTCCAG
32a-SAH-F	TACCGACGACGACGACAAGGCCATGATGTTCAGCTTTGGCGGC
32a-SAH-R	TTCGGGCTTTGTTAGCAGCCGGATCTTAGTGGTGATGGTGATGAT GTTCCA
32a-SSH-F	TACCGACGACGACGACAAGGCCATGATGACGTTTGCGTACAAGA TCG
32a-SSH-R	TTCGGGCTTTGTTAGCAGCCGGATCTTAGTGGTGATGGTGGTGAT GCA
pMC68-F	GCTAGCTTGGTACGTACCAGATC
pMC68-R	ACCAAGATCATTTCCGTCATATGCATAC
pMC68-ARS-F	TGCATATGACGGAAATGATCTTGGTGTTCCGGCAAGCACATATAG AC
pMC68-ARS-R	CAGATCTGGTACGTACCAAGCTAGCTTAGTGATGATGATGATGAT GTGTTTCGAC
pMC68-ARH-F	TGCATATGACGGAAATGATCTTGGTATGAATCGCAGATTTCCAGTT TGG
pMC68-ARH-R	CAGATCTGGTACGTACCAAGCTAGCTTAGTGATGGTGATGATGAT GTTCCAG
pHT43-F	AGCCCGCCTAATGAGC
pHT43-R	TGATCCTTCCTCCTTTAATTGGGAAT
pHT43-CBM68-F	TTCCCAATTAAAGGAGGAAGGATCAATGCCCCCAAAACAACAGT CG

pHT43-CBM68-R	GCAGTCTATATGTGCTTGCCGGAACACCAAGATCATTTCCGTCATATGCATAC
pHT43-ARS-F	TGCATATGACGGAAATGATCTTGGTGTTCCGGCAAGCACATATAGAC
pHT43-ARS-R	CGCTCATTAGGCGGGCTGCCCCGGGTTAGTGATGATGATGATGATGTGTTTCGAC
pHT43-ARH-F	TGCATATGACGGAAATGATCTTGGTATGAATCGCAGATTTCCAGTTTGG
pHT43-ARH-R	CGCTCATTAGGCGGGCTGCCCCGGGTTAGTGATGGTGATGATGATGTTCCAG
pDG1730-F	GGGCAAGGCTAGACGGGAC
pDG1730-R	TCTTGACACTCCTTATTTGATTTTTTG
1730-amyE-up-F	CAAAAAATCAAATAAGGAGTGTCAAGAATGTTTGCAAAACGATTCAAAACCTC
1730-amyE-up-R	AATCAGCAAGGGACAGGTAGTACCGAAGCTTCTAGGATCCGATCAGACCAG
Hpa II-F	TACTACCTGTCCCTTGCTGATTTTT
Hpa II-R	GATCTGCATCCGCTTACAGACA
Spc-F	CTGTAAGCGGATGCAGATCATCGAATTCCTGCAGCCCTG
Spc-R	GATCCCCCTATGCAAGGGTTTATTG
1730-amyE-down-F	CAATAAACCCTTGCATAGGGGGATCTCGACATGGATGAGCGATGATG
1730-amyE-down-R	GTCCCGTCTAGCCTTGCCCTCAATGGGGAAGAGAACCGCTTAAG
1730-nprB-up-F	CAAAAAATCAAATAAGGAGTGTCAAGAGTTTTCCCTTAAAACAAATATTTGAACACGATTTTGGC
1730-nprB-up-R	AATCAGCAAGGGACAGGTAGTACCGGCAAACAAAAACAGTCAGGACA
1730-nprB-down-F	CAATAAACCCTTGCATAGGGGGATCATGAAAACGAATCAAGTTAATGACCGC
1730-nprB-down-R	GTCCCGTCTAGCCTTGCCCAACACCACATCCTTCCTATTTTGGAATCTACC
1730-ytxE-up-F	CAAAAATCAAATAAGGAGTGTCAAGACTGACCTCTTTCTTCATCGTAACAGG
1730-ytxE-up-R	AATCAGCAAGGGACAGGTAGTACCGTGCAGATCGATTTGGG
1730-ytxE-down-F	CAATAAACCCTTGCATAGGGGGATCGGATAACAAGACAAATGAACACATGAAGG
1730-ytxE-down-R	GTCCCGTCTAGCCTTGGAGAGCATCAGGATTCTGCATATACA

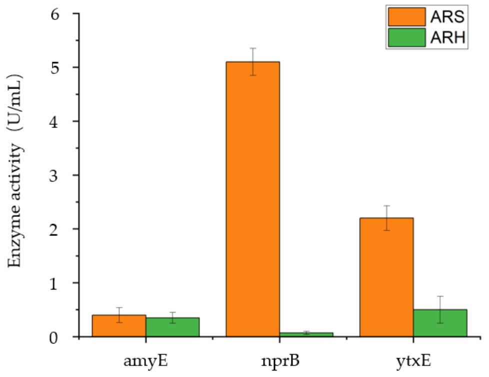

(A)

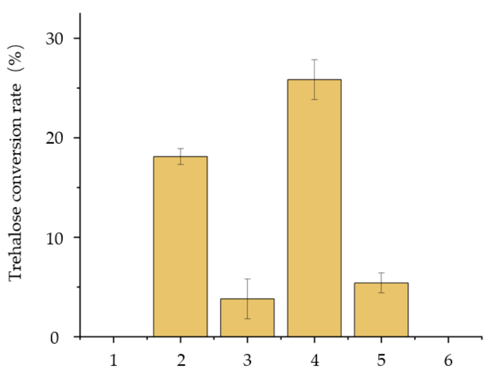

(B)

Figure S1. Expression of ARS and/or ARH by genome integration in *B. subtilis* SCK6. A. Enzyme activities of the recombinant ARS or ARH by genome integration in different positions of *B. subtilis* SCK6 genome. B. Trehalose conversion rates of the extracellular multi-enzyme produced by different co-expression genome integration strains. 1: the genome integration strain that inserted encoding genes of ARS and ARH into *amyE* and *nprB* locations of the genome, respectively; 2: the genome integration strain that inserted encoding genes of ARS and ARH into *amyE* and *ytxE* locations of the genome, respectively; 3: the genome integration strain that inserted encoding genes of ARS and ARH into *nprB* and *amyE* locations of the genome, respectively; 4: the genome integration strain that inserted encoding genes of ARS and ARH into *nprB* and *ytxE* locations of the genome, respectively; 5: the genome integration strain that inserted encoding genes of ARS and ARH into *ytxE* and *amyE* locations of the genome, respectively; 6: the genome integration strain that inserted encoding genes of ARS and ARH into *ytxE* and *nprB* locations of the genome, respectively.

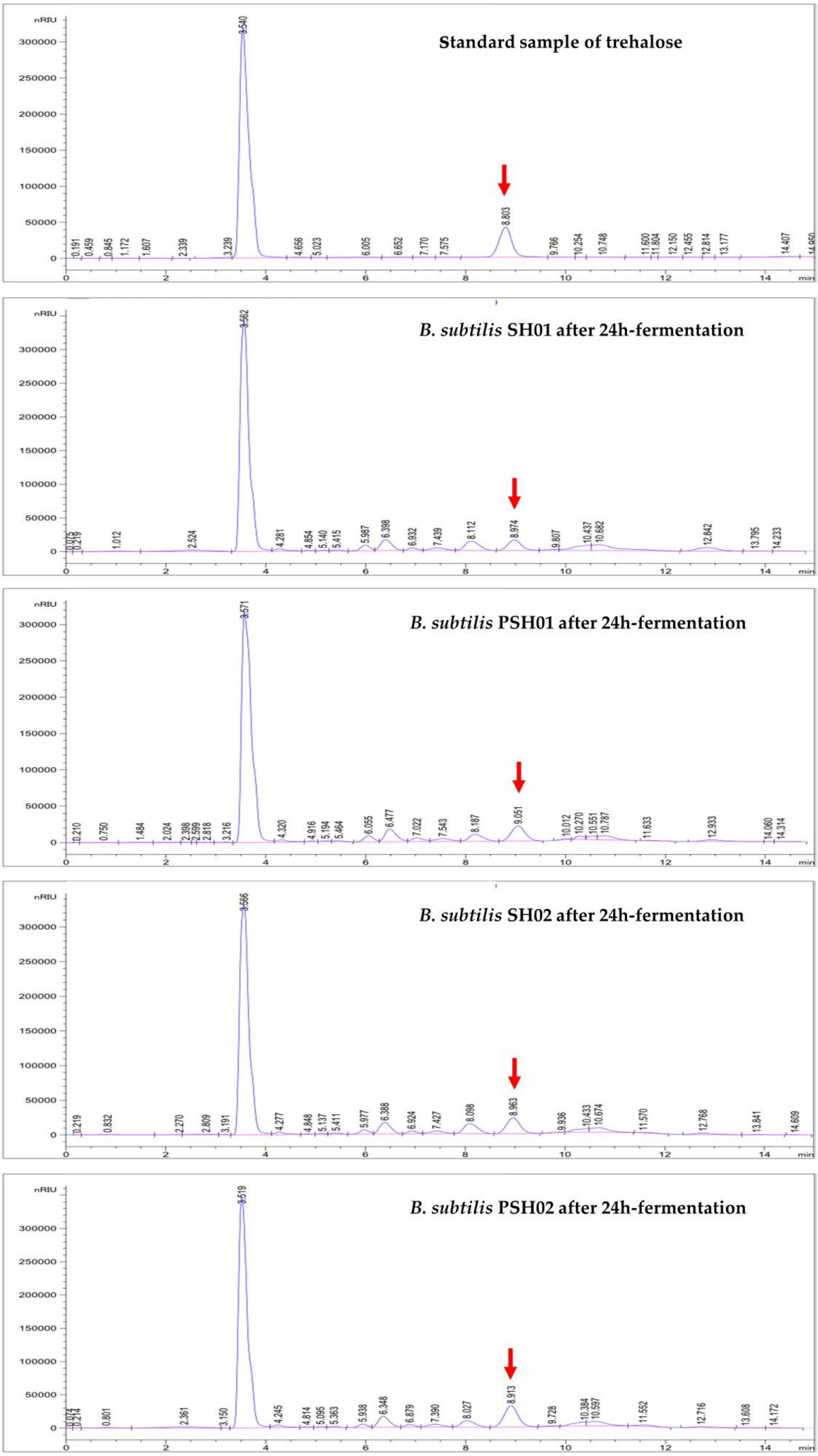

Figure S2. The HPLC chromatograms of extracellular enzymes solutions produced constructed strains. Chromatogram of trehalose standard sample with a concentration of 10

mg/mL. The detection peak was shown at 8.803 min in the topside map. Using 10 mg/mL maltodextrin as the substrate, extracellular enzymes solutions after 24 h cell-fermentations were prepared and added for reactions. Productions were detected and the chromatogram maps were presented in the order of strains *B. subtilis* SH01, PSH01, SH02, PSH02 from top to bottom with determination peak of 8.974 min, 9.051 min, 8.963 min, 8,913 min correspondingly.

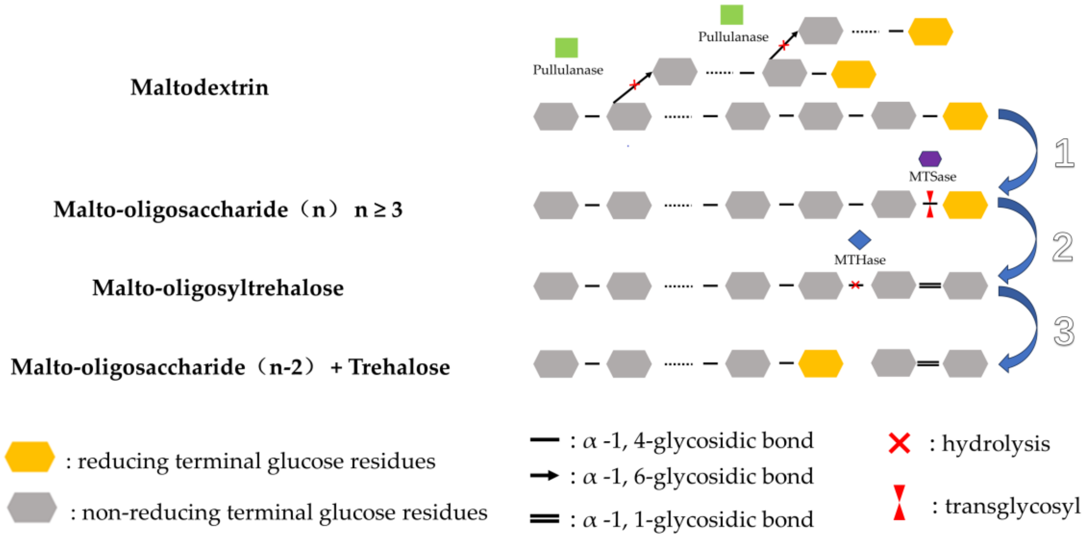

Figure S3. Converting pathway from maltodextrin to trehalose by double enzymes system. In step 1, pullulanase was added as an assistant enzyme to recognize and cut the α-1,6 glycosidic bond of maltodextrin. This reaction created more reducing ends to be transglycosylated by MTSase in step 2 and produced malto-oligosyltrehalose to be the substrate for next step. In step 3, MTHase hydrolysised the penult end of the long chain, resulting Trehalose and malto-oligosyltrehalose chain lacking two glycosyl groups.
